# Unlocking innovation and resilience among emergency nurses through cultural intelligence: insights from a structural equation model

**DOI:** 10.1186/s12912-025-03569-w

**Published:** 2025-07-15

**Authors:** Nadia Hassan Ali Awad, Boshra Karem Mohamed El-Sayed, Heba Mohamed Al-Anwer Ali Ashour

**Affiliations:** Nursing Administration Department, Faculty of Nursing, Alexandria University, Alexandria, Egypt

**Keywords:** Cultural intelligence, Cultural quotient, Emergency nurses, Innovative work behavior, Resilience

## Abstract

**Background:**

The dynamic, multicultural healthcare environment and increasing worker diversity highlight the importance of cultural intelligence (CQ). High cultural intelligence helps employees seek colleague assistance, enhances resilience, and encourages innovative behavior. This study aims to develop a structural equation model (SEM) to test the impact of culture intelligence as a mediating factor between resilience and innovative work behavior (IWB) among emergency care nurses.

**Methods:**

A convenience sample of 276 nurses from four emergency departments in Alexandria participated in a cross-sectional, correlational study. Three validated scales were used to measure the study variables.

**Results:**

The study found that nurses had moderate mean scores for culture intelligence (58.94%), resilience (58.40%), and innovative work behavior (61.49%). The structural equation model analysis showed a good fit (CFI = 1.000, IFI = 1.000, RMSEA = 0.063), confirming that culture intelligence mediates the relationship between resilience and innovative work behavior, with a p-value less than 0.05.

**Conclusions:**

The study highlights the significant influence of cultural intelligence on resilience and innovative work behavior in emergency nurses. The findings contribute to the growing literature by establishing a model linking these variables, emphasizing the role of cultural intelligence in enhancing resilience and creativity in high-pressure settings. Human resource managers should implement cultural intelligence-focused educational programs and revise recruitment criteria to select nurses with high cultural intelligence traits.

**Clinical trial number:**

Not applicable.

## Background

In today’s dynamic and multicultural healthcare environment, nurses, especially those in Emergency Rooms (ERs), interact continuously with patients from diverse backgrounds. This increases the interest in Cultural Intelligence (CQ), which enhances cross-cultural understanding and practical skills in healthcare settings. One key skill associated with CQ is resilience, which helps nurses manage workplace challenges. Resilient nurses with high CQ adapt more effectively, respond creatively to adversity, and demonstrate greater perseverance. Both CQ and resilience contribute to problem-solving, adaptability, and ultimately fostering innovative work behavior (IWB), which is essential for innovation and high performance in demanding healthcare environments [1–3].

## Theoretical framework

Social Cognitive Theory (SCT) explains how cultural intelligence (CQ) and resilience contribute to innovative work behavior (IWB) through the reciprocal interaction of personal, environmental, and behavioral factors. In emergency nursing, CQ helps nurses navigate diverse cultural interactions, improving communication and patient trust, while resilience enables them to adapt under pressure and maintain effectiveness in high-stress situations. SCT suggests that exposure to multicultural challenges strengthens CQ and resilience over time, reinforcing problem-solving and adaptability.

**Fig. 1:**
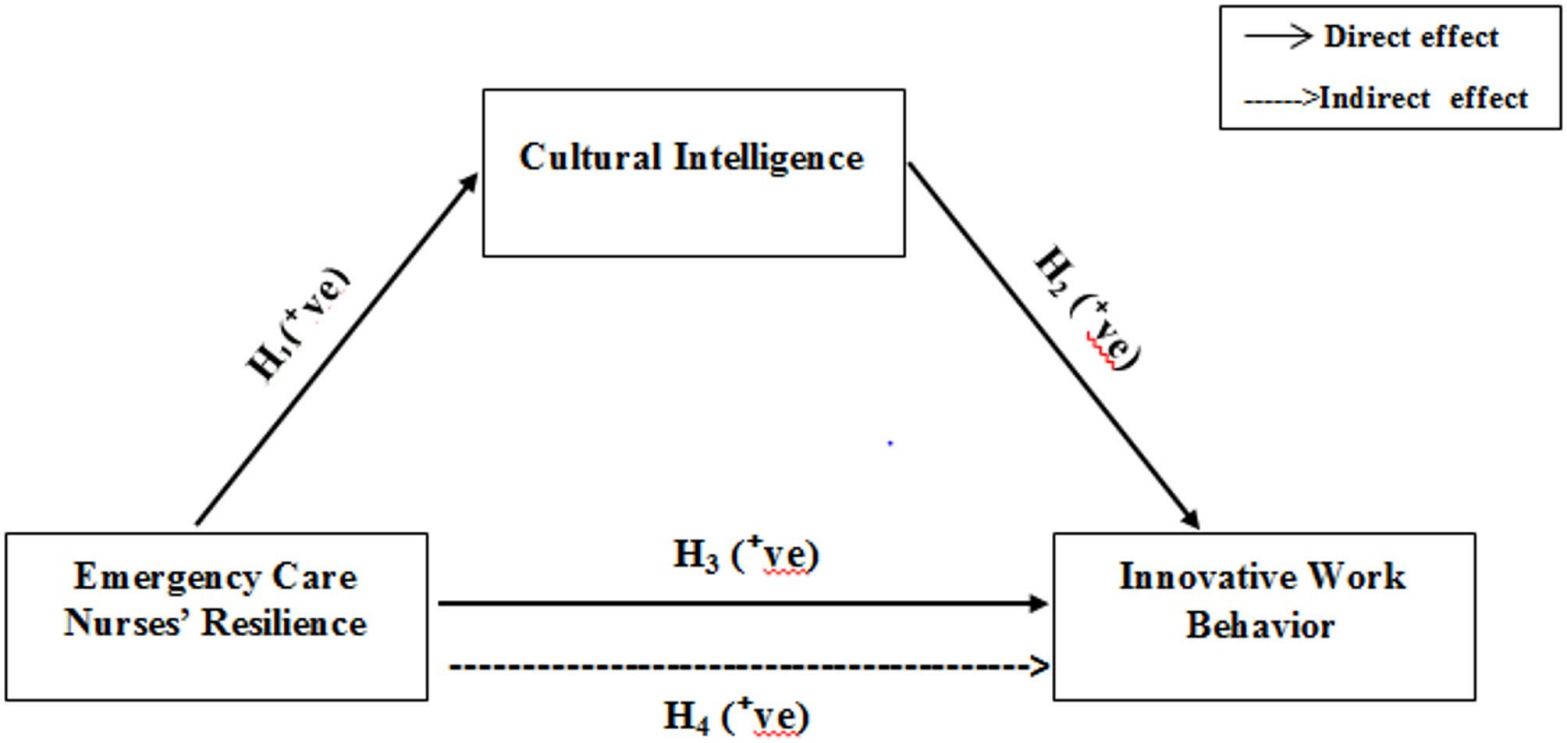
Conceptual model of the study

High CQ fosters creativity and culturally responsive care, while resilience supports persistence in implementing new ideas. These qualities drive innovation in emergency settings, where quick decision-making and adaptability are crucial. Due to its demanding nature, emergency nursing is an ideal context for studying these relationships. Research highlights the impact of multicultural competence on patient outcomes and the necessity of resilience in managing workplace challenges. Understanding how CQ and resilience influence IWB can help enhance nurse performance and healthcare quality in diverse settings. Based on that, the researchers considered this theory to build their conceptual framework and explain how cultural intelligence may influence emergency care nurses’ resilience and innovative work behavior [4–8]. Please, see Fig. 1.

### Cultural intelligence/ cultural quotient (CQ)

Emergency Nurses (ERNs) are crucial for patient care, managing pain, facilitating recovery, and interacting with individuals from diverse backgrounds. To provide effective care, ERNs must develop cultural intelligence (CQ) to navigate cross-cultural interactions successfully [9]. Cultural intelligence is defined by Ang and Van Dyne (2015) as the ability to function effectively in culturally diverse environments. Unlike general cultural competence, CQ is not limited to understanding specific cultures but reflects an individual’s adaptability across various cultural contexts [9–13]. Existing research has explored CQ’s role in improving communication and patient care; however, limited studies have examined its function in enhancing resilience and fostering innovative work behavior (IWB) among nurses. The connection between CQ and these workplace dynamics remains underexplored, particularly in high-pressure environments like emergency care [14, 15].

Cultural intelligence includes four key dimensions: metacognitive, cognitive, motivational, and behavioral. The **metacognitive** and **cognitive** dimensions relate to cultural awareness and understanding, while the **motivational** and **behavioral** dimensions reflect an individual’s willingness and ability to adapt in cross-cultural settings [10]. Beyond its role in improving patient outcomes, CQ may serve as a critical mediating factor between resilience and IWB. Resilience enables nurses to manage workplace challenges, but its impact on innovation depends on their ability to engage effectively in diverse teams and adapt to changing scenarios. CQ enhances this adaptability by fostering cognitive flexibility, reducing biases, and promoting collaboration. This cultural adaptability facilitates innovative problem-solving and creative decision-making in healthcare settings [16–19]. By examining CQ as a bridge between resilience and IWB, this study aims to fill a crucial literature gap and offer insights into how ERNs can develop their personal and professional competencies to enhance patient care and workplace efficiency.

### Resilience at work

Resilience at work is defined by Malik and Garg (2018) as an individual’s ability to manage challenging circumstances and the daily stress of work while maintaining their health, bounce back from setbacks, and learn from them [20]. It also involves proactively preparing for future challenges and demonstrating increased competence, professional growth, and the capacity to handle future challenges in the workplace. Delgado et al. (2020) defined it as selfhood, flexibility, faith, self-confidence, and creativity, an empathic and humanistic approach, and developed insight about responsibilities and roles, good physical health to contribute to mental wellness, good social network, and hobbies [21]. Therefore, resilience is the ability of the individual to adapt and thrive in a challenging environment. Winwood et al. (2013) found that resilience is built upon seven domains: living authentically, finding your calling, maintaining perspective, managing stress, interacting cooperatively, staying healthy, and building networks [20, 22].

Using one’s strengths, adhering to reasonable emotional awareness and moderation, and living a true life are the foundations of *authentic living*. *Finding your calling* is associated with having a job that provides meaning, fosters community, and synchronizes work with personal fundamental principles and convictions [22, 23]. Reframing failures, reducing the influence of negativity around you, and being upbeat and solution-focused when things go wrong are all ways to *maintain perspective*. *Managing stress* is achieved through treating common stressors, striking a work-life balance, and making time for rest. Another domain of resilience at work *is interacting cooperatively* while seeking feedback, advice, and support. Maintaining a normal weight, eating balanced food, and getting enough sleep are all important components of *staying healthy*. Establishing and sustaining professional and personal support networks to *build networks* and do a good job at work [24].

Rezaei et al. (2021) elucidated that resilient personnel possess superior mental health, self-regulation abilities, and elevated self-esteem. Additionally, they exhibit greater familial support and fewer hazardous behaviors [25]. Nurses with resilience are better able to control their emotions and form strong bonds with others. It is necessary to help nurses adapt to nursing work’s physical and emotional needs, calmly deal with various difficulties and adversity, and alleviate the helpless mood during challenging times. In addition to improving general well-being, quality of life, and job satisfaction, high resilience in nursing lowers job stress, burnout, compassion fatigue, and turnover intent [26, 27]. Building resilience enables nurses to achieve occupational well-being and professional advantages, paving the way for their success on a personal and professional level [28]. Chadwick and Raver (2020) and Malik and Garg (2020) reported a positive relationship between resilience and innovative work behavior [29, 30].

### Innovative work behavior (IWB)

Healthcare organizations face more obstacles in this ever-changing world, and for nurses to provide their services, maintain their competitiveness, and drive change, they must encourage innovative behaviors. Alessa and Durugbo (2021) defined innovating work behavior (IWB) as a complex self-initiated multi-dimensional behavior that nurses intentionally generate, introduce, and apply through critical thinking, identifying opportunities and solutions, recognizing existing and potential problems, identifying performance gaps, and seeking new methods and procedures to improve organizational performance both internally and externally to create value, gaining a competitive advantage, and ensuring sustainability [31, 32].

Lukes and Stephan (2017) classified IWB into seven dimensions: idea generation, idea search, idea communication, implementation starting activities, involving others, overcoming obstacles, and innovation outputs. *Idea generation* refers to the stage during which nurses recognize problems and generate innovative solutions to deal with these problems. *Idea search* is an activity that may also be promoted by individuals searching for new ideas based on searches of existing knowledge sources in their environment. *Idea communication* is the communication of ideas with managers, receiving their feedback, and gaining their permission. *Implementation of activities* starts with problem anticipation, proactively developing contingency plans, and acquiring resources and funds to resolve the issue.

*Involving others* is an innovation champion who focuses on involving others in the implementation, communicates a vision of what the innovation entails, and displays confidence and enthusiasm about it. *Overcoming obstacles*,* barriers*,* and resistance* is achieved by adapting the idea or implementation plans until the improvement of service or process is achieved in the organization. Finally, *innovation outputs* are the outcomes of implementing the innovation and are represented through achieved change reports within the organization [32].

### Role of culture intelligence (CQ) as a mediation factor

Nurses with high cultural intelligence (CQ) can effectively leverage their resilience to deliver more innovative patient care. While resilience helps them navigate stress and challenges, CQ enhances their ability to understand diverse cultural backgrounds, improving teamwork and communication. This combination allows them to develop creative solutions, such as tailoring treatments to align with cultural norms or fostering collaboration in diverse healthcare settings. Moreover, CQ supports leadership, ethical decision-making, and the effective use of advanced medical technology in multicultural environments. By integrating resilience with cultural intelligence, nurses can adapt more efficiently, address challenges, and implement innovative approaches that improve patient care and overall healthcare outcomes [33, 34].

### Significance of the study

Emergency care nurses (ERNs) or critical care nurses operate in fast-paced, high-pressure environments characterized by time constraints, heavy workloads, and numerous responsibilities. These challenges are further intensified by limited resources, exposure to human suffering, and the need to maintain an error-free practice. They frequently interact with patients, colleagues, and healthcare providers from diverse cultural backgrounds while adapting to rapidly evolving medical technologies. These demanding conditions affect their mental health, professionalism, quality of care, financial stability, resilience, and innovative work behavior (IWB), and even contribute to increased turnover and resignation rates [3]– [4, 9, 35]– [36]. Conversely, fostering innovative work practices has become essential for enhancing organizational effectiveness and maintaining a competitive edge. Research suggests that workplace creativity and resilience are closely linked to cultural intelligence (CQ), highlighting its critical role in shaping adaptive and innovative behaviors in dynamic healthcare settings [37, 38].

The American Nurses Association (2015) introduced the *Nursing Scope and Standards of Practice: Standard 8* to ensure that registered nurses provide culturally congruent care that aligns with principles of diversity and inclusion. This standard emphasizes the importance of addressing the needs of patients from diverse cultural backgrounds. The association called for further research to support the integration of culturally congruent practices within nursing. Given the increasingly diverse nursing workforce, leaders should develop and demonstrate cultural intelligence (CQ) to navigate effectively and support cross-cultural interactions in healthcare settings [39].

Extensive international research has examined cultural intelligence among nurses, nurse practitioners, and nursing students [40, 41]. Previous studies have explored the relationships between cultural intelligence, innovative work behavior (IWB), and resilience, but there remains a significant gap in understanding how cultural intelligence mediates these dynamics [42, 43]. In Egypt, some research has reported unsatisfactory levels of cultural intelligence, resilience, and IWB among emergency nurses among nurses. However, the extent to which cultural intelligence strengthens or facilitates the connection between resilience and IWB, particularly in diverse organizational settings, remains largely unexplored [44, 45].

Addressing this gap is essential to understanding how resilience contributes to innovation and how individuals navigate cultural complexities while fostering innovation in the workplace. The lack of evidence on the role of cultural intelligence in shaping critical care nurses’ resilience and innovative behaviors is concerning. This study aims to bridge this gap by examining the influence of cultural intelligence on resilience and IWB among emergency nurses. Gaining insights into these relationships is crucial for designing effective interventions and developing organizational policies that enhance cultural intelligence, resilience, and innovation among nurses and healthcare leaders.

### Aim of this study

This study aims to examine the relationships among cultural intelligence, resilience, and innovative work behavior among emergency care nurses. Specifically, it seeks to investigate the mediating role of cultural intelligence in the relationship between nurses’ resilience and their engagement in innovative work behavior.

### Research questions and hypotheses

#### RQ1

What are the relationships among cultural intelligence, emergency care nurses’ resilience, and innovative work behavior?

#### RQ2

Does cultural intelligence mediate the relationship between resilience and innovative work behavior?

### Research hypotheses

#### H_1_

Emergency care nurses’ resilience is significantly and positively associated with their cultural intelligence.

#### H_2_

Cultural intelligence is significantly and positively associated with emergency care nurses’ innovative work behavior.

#### H_3_

Emergency care nurses’ resilience is significantly and positively associated with their innovative work behavior.

#### H_4_

Cultural intelligence significantly mediates the relationship between resilience and innovative work behavior.

## Methods

### Research design and setting

Three validated measures were used to assess the study variables in a cross-sectional and correlational investigation based on the conceptual framework. Four emergency rooms connected to three general governmental hospitals in Alexandria were selected for this study. They were chosen based on the number of nurses, bed capacity, and a somewhat comparable service offered.

### Participant

A non-probability convenience sampling method was used to recruit 276 full-time emergency care nurses from four institutions, with approximately 69 nurses from each hospital. Participants were eligible if they had at least six months of clinical experience and voluntarily agreed to participate. The total sample size was determined using Epi-Info, based on a total nurse population (*N* = 470), an acceptable error of 5%, a significance level (**α** = 0.05), and a confidence coefficient of 99%.

This convenience sampling method was chosen due to practical considerations, including its feasibility in reaching enough emergency nurses within the study timeframe and allowing efficient data collection while ensuring representation across multiple institutions. However, this approach has limitations, including potential selection bias and limited generalizability beyond the sampled institutions.

### Instruments

In addition to the demographic and work-related characteristics of the study subjects, such as gender, age, educational background, and years of experience in the current working unit, three standardized questionnaires were used to gather the necessary data through self-reported questionnaires.

#### Tool (I): cultural intelligence scale (CQS)

To evaluate emergency room (ER) nurses’ perceptions of cultural intelligence (CQ), a self-administered questionnaire grounded in the CQ framework was employed. This tool was originally developed by Ang et al. (2007) and subsequently adapted by Barzykowski et al. (2019). It consists of 20 items categorized into four distinct dimensions: cognitive (6 items), metacognitive (4 items), behavioral (5 items), and motivational (5 items). Participants responded using a 5-point Likert scale ranging from 1 (strongly disagree) to 5 (strongly agree), yielding a total possible score between 20 and 100. Higher scores reflect a greater perceived level of cultural intelligence. The instrument demonstrated excellent internal reliability, with a Cronbach’s alpha of 0.95 [46, 47].

#### Tool (II): resilience at work scale (RAW-S)

The Resilience at Work Scale, developed by Winwood et al. (2013), was employed to assess resilience levels among emergency room (ER) nurses within the workplace. The scale comprises 20 items distributed across seven dimensions: living authentically (3 items), finding your calling (4 items), maintaining perspective (3 items), managing stress (4 items), interacting cooperatively (2 items), staying healthy (2 items), and building networks (2 items). Responses are rated on a 5-point Likert scale ranging from 1 (strongly disagree) to 5 (strongly agree), with total scores ranging from 20 to 100. Higher scores reflect greater perceived resilience at work. The instrument demonstrated strong internal reliability, with a Cronbach’s alpha of α = 0.90 [22].

#### Tool (III): innovative work behavior scale (IWBS)

It was developed by Lukes and Stephan (2017), was utilized to assess the extent of creative and innovative behaviors demonstrated by emergency room (ER) nurses in the workplace. The scale consists of 23 items categorized into seven dimensions: idea generation (3 items), idea search (3 items), idea communication (4 items), implementation-starting activities (3 items), involving others (3 items), overcoming obstacles (4 items), and innovation outputs (3 items). Responses were measured using a 5-point Likert scale ranging from 1 (strongly disagree) to 5 (strongly agree). Total scores range from 23 to 115, with higher scores indicating greater levels of innovative work behavior. The instrument demonstrated acceptable internal reliability, with a Cronbach’s alpha of 0.81 [32].

### Validity and reliability of translated tools

Ensuring the accuracy and applicability of the study instruments in the Egyptian healthcare setting required a rigorous translation, validation, and reliability assessment process. The following steps were undertaken:

#### Translation and cultural adaptation

The original study tools were translated into Arabic by two independent bilingual healthcare experts to ensure linguistic and conceptual accuracy. The translated versions were adapted to conform to Egyptian cultural norms and healthcare terminology, ensuring relevance and clarity for emergency nurses. Then, a back-translation was performed by an independent translator with no prior knowledge of the original tools [48]. This process aimed to identify discrepancies and confirm that the Arabic version accurately reflected the original content. Any inconsistencies between the original and back-translated versions were reviewed and resolved through a consensus approach involving linguistic and subject-matter experts.

#### Validity and reliability testing

A panel of five academic experts (four professors and one assistant professor of nursing administration) evaluated the Arabic version of the instruments for validity and translation fluency. The validity focused on ensuring that each item adequately measured the intended construct (cultural intelligence, resilience, and innovative work behavior). However, validity assessed whether the items were clear, unambiguous, and appropriate for the target population. Experts provided feedback, leading to minor modifications to enhance clarity and cultural relevance. In addition, a pilot study was conducted on 10% of emergency room nurses (*n* = 27) and excluded from the study population to verify the accuracy and determine the time required to complete the questionnaire.

A reliability analysis was employed to assess the internal consistency of the indicators of the underlying factor. The reliability of study instruments was assessed using Cronbach’s alpha. The Cultural Intelligence scale, Resilience at Work scale, and Innovative Work Behavior scale had overall coefficient alpha values of 0.886, 0.926, and 0.862, respectively, indicating strong internal consistency.

### Data collection

Written approval was obtained from the Faculty of Nursing at Alexandria University’s Research Ethics Committee before the study could begin. Furthermore, authorizations from the administrative body in charge of the study setting were acquired. The participants were informed of the study’s goal, advised that their information would be kept confidential, and given their informed consent to participate. The researchers then collected data through hand-delivered anonymous questionnaires to the study subjects. Completing the questionnaire required an average of fifteen to twenty minutes. Data was collected over three months.

### Statistical analysis

Cronbach’s alpha was employed to evaluate the reliability of the tools. Frequency and percentage were used to assess the participants’ demographic information. Mean score and standard deviation were used to define the study variables. A Pearson’s correlation test was used to investigate the relationships between the research variables. Using structural equation modeling (SEM) and AMOS Ver. 23, the impact of culture intelligence as a mediating factor on the resilience and innovative work behavior of ER nurses was examined. The model fit indices included the evaluation of; χ2/df = Chi Square/degree of freedom, CFI = Comparative fit index; IFI = Incremental Fit Index; RMSEA = Root Mean Square Error of Approximation; NFI = Normed fit index; RFI = Radio Frequency Interference; NNFI (Non-Normed Fit Index); SRMR (Standardized Root Mean Square Residual); GFI (Goodness of Fit Index); AGFI (Adjusted Goodness of Fit); TLI (Tucker-Lewis Index).

## Results

**Table 1:** Distribution of ERNs according to demographic and work-related characteristics (*n* = 276)

Demographic and work-related characteristics	No	%
Age		
< 30	39	14.13
30–40	139	50.36
41–50	79	28.62
> 50	19	6.89
Age (X ± SD)	35.5 ± 10.154	
Gender		
Male	104	37.7
Female	172	62.3
Educational level		
Technical Secondary School of Nursing	86	31.2
Technical Institute Diploma in Nursing	68	24.61
Nursing bachelor’s degree	119	43.11
Master’s degree	3	1.08
Years of experience in the nursing profession		
< 5	8	2.9
5–10	13	4.7
11–20	140	50.7
> 20	115	41.7
Mean ± SD	14.04 ± 7.54	
Years of experience in the ER unit		
< 5	8	2.89
5–10	82	29.72
11–20	152	55.07
> 20	34	12.32
Mean ± SD	13.59 ± 8.39	

Table 1 shows the distribution of ER nurses regarding their demographic and work-related characteristics; the mean score of ER nurses’ ages was 35.5 ± 10.154. Also, 62.3% of them were females. 43.11% hold a bachelor’s degree in nursing science, and 31.2% have a Diploma from the Technical Secondary School of Nursing. The mean score of nurses’ years of work experience in the nursing profession was (14.04 ± 7.54), the highest percentage of nurses (50.7%) had experience from 11 years to less than 20 years, and 41.7% had more than 20 years of work experience in nursing. According to the mean score of nurses’ years of work experience in the ER unit was (13.59 ± 8.39), more than half of ER nurses (55.07%) had experience from 11 years to less than 20 years, and 29.72% had 5–10 years of work experience as ER nurses.

**Table 2:** The overall mean score of perceived cultural intelligence, resilience at work, and innovative work behavior (*n* = 276)

Variables	Min-Max	Mean ± SD	Mean percent score
Cognitive	11.00–29.00	19.78 ± 3.03	57.41
Metacognitive	7.00–20.00	13.62 ± 3.09	60.11
Behavioral	8.00–25.00	17.07 ± 2.90	60.33
Motivational	9.00–25.00	16.69 ± 3.00	58.45
**Overall Cultural Intelligence**	**47.00–94.00**	**67.15 ± 9.08**	**58.94**
Living authentically	6.00–15.00	9.93 ± 2.07	57.79
Finding one’s calling	7.00–20.00	13.35 ± 2.21	58.45
Maintaining perspective	5.00–15.00	9.95 ± 2.28	57.91
Managing stress	7.00–20.00	13.43 ± 2.43	58.91
Interacting cooperatively	2.00–10.00	6.81 ± 1.38	60.14
Staying healthy	2.00–10.00	6.68 ± 1.63	58.45
Building networks	2.00–10.00	6.57 ± 1.53	57.14
**Overall Resilience at Work**	**44.00–94.00**	**66.72 ± 8.67**	**58.40**
Idea generation	6.00–15.00	10.73 ± 2.02	64.45
Idea search	3.00–15.00	10.79 ± 2.80	64.94
Idea communication	6.00–20.00	14.36 ± 3.35	64.73
Implementation of starting activities	5.00–15.00	10.31 ± 1.79	60.91
Involving others	3.00–15.00	9.99 ± 2.07	58.24
Overcoming obstacles	6.00–20.00	13.47 ± 2.38	59.20
Innovation outputs	3.00–15.00	9.92 ± 2.04	57.67
**Overall Innovative work behavior**	**55.00–99.00**	**79.57 ± 8.60**	**61.49**

Table 2 indicates that the majority of emergency room nurses (ERNs) demonstrated moderate levels of cultural intelligence, workplace resilience, and innovative work behavior. These perceptions were reflected across all measured dimensions, with mean percentage scores of 58.94% for cultural intelligence, 58.40% for resilience, and 61.49% for innovative work behavior.

**Table 3:** Pearson correlations matrix between the study variables (*n* = 276)

		Cultural Intelligence	Resilience at Work	Innovative Work Behavior
Cultural Intelligence	r		0.604	0.588
	p		< 0.001*	0.002*
Resilience at Work	r	0.504		0.664
	p	< 0.001*		< 0.001*

r: Pearson coefficient

*: Statistically significant at *p* ≤ 0.05

Table 3 revealed statistically significant positive correlations among the key study variables. Cultural intelligence was positively associated with both resilience at work (*r* = 0.504, *p* < 0.001) and innovative work behavior (*r* = 0.588, *p* = 0.002) among emergency room nurses. Additionally, a strong and statistically significant positive correlation was found between resilience at work and innovative work behavior (*r* = 0.664, *p* < 0.001).

**Table 4:** Path analysis direct and indirect effect of resilience at work on innovative work behavior mediated by cultural intelligence

Path	Estimate	*R*²	SE	C.*R*.	*p*-value
CQ ← Resilience at Work	0.304	0.318	0.060	5.282*	< 0.001*
IWB ← Resilience at Work (Direct Effect)	0.228	0.226	0.060	3.759*	< 0.001*
IWB ← Cultural Intelligence	0.119	0.113	0.058	1.960*	0.049*
**Indirect Effect (Resilience **→ **CQ **→ **IWB)**	**0.036**	—	—	—	0.002*

CFI = Comparative fit index; IFI = incremental fit index; and RMSEA = Root Mean Square Error of Approximation

Model fit: CFI = 1.000, IFI = 1.000, RMSEA = 0.063

Model Chi-square (X²) = 16.749, *p* < 0.001

**Fig. 2:**
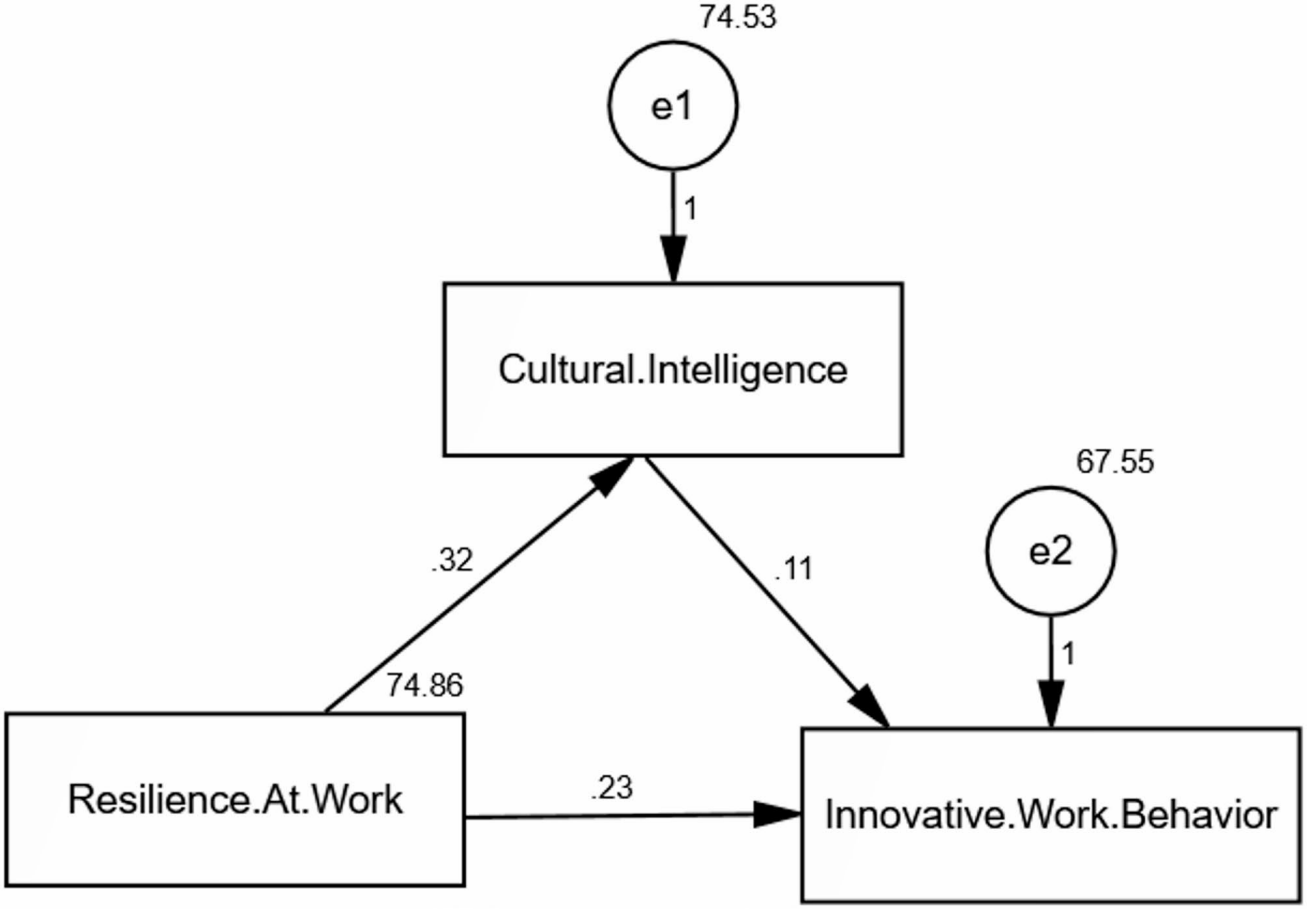
Path analysis direct and indirect effect of resilience at work on innovative work behavior mediated by cultural intelligence

Table 4; Fig. 2 present the results of the path analysis examining both the direct and indirect effects of resilience at work on innovative work behavior (IWB) with cultural intelligence (CQ) as a mediator. Resilience was found to have a significant direct effect on both CQ (β = 0.304, *p* < 0.001) and IWB (β = 0.228, *p* < 0.001). CQ also had a significant direct effect on IWB (β = 0.119, *p* = 0.049). The computed indirect effect of resilience on IWB through CQ was β = 0.036, suggesting a small but statistically significant mediating effect. These findings confirm partial mediation, indicating that while CQ mediates the relationship between resilience and IWB, resilience also has a direct influence on IWB. The model demonstrated excellent fit indices (CFI = 1.000, IFI = 1.000, RMSEA = 0.063), supporting the adequacy of the hypothesized model.

## Discussion

Healthcare institutions are rapidly evolving in response to global health challenges, the constant integration of new medical knowledge, and the imperative for cost-effective care delivery [49]. These shifts are increasingly situated in culturally diverse environments, where healthcare professionals must operate effectively despite cultural differences. In this context, cultural intelligence (CQ) becomes a vital competency [50–52]. The findings of this study revealed that ER nurses demonstrated a moderate level of CQ across their four dimensions: cognitive, metacognitive, behavioral, and motivational. This may stem from their frequent interactions with patients from varied cultural backgrounds, combined with the high-paced nature of emergency settings that limit time for culturally tailored communication. However, the moderate levels may also reflect a lack of institutional training on intercultural competencies. This aligns with Ahanchian et al. (2012) and other studies [53], who reported similar moderate CQ levels among nurses. In contrast, Presbitero (2016) and Berhanu et al. (2024) emphasized low CQ levels, urging the integration of structured educational interventions [54, 55].

Resilience, another core construct of this study, is essential for sustaining psychological and emotional functioning in high-stress environments. Emergency nurses frequently encounter occupational stressors such as time pressure, emotional exhaustion, and moral dilemmas [44]. The study’s finding of moderate resilience is consistent with literature by Kyranou et al. (2025), Kannappan and Veigas (2021), and Delgado et al. (2017) [56–58]. These findings suggest that although ER nurses manage to adapt, they remain at risk of burning out. Contrastingly, the studies of Sam and Lee (2020), Oliveira et al. (2020) have reported lower resilience levels [59, 60], while O’Doherty et al. (2024), Chow et al. (2018), and Chamberlain et al. (2016) found higher resilience scores, potentially may be due to contextual or institutional factors [61–63].

Innovative Work Behavior (IWB), which includes idea generation, promotion, and implementation, is increasingly recognized as a key driver of organizational adaptability [64]. ER nurses reported moderate IWB levels, possibly due to limited exposure to innovation-focused training and support systems. This is consistent with Abd El-Fattah (2017) and Jung and Yoon (2018) [65, 66], who noted that innovation remains underdeveloped in routine nursing practice. In contrast, Ahmed et al. (2019) and Li et al. (2025) reported higher levels of IWB among nurses, suggesting that managerial encouragement and organizational culture significantly influence innovative engagement [49, 67].

Importantly, this study confirmed a statistically significant positive correlation among CQ, resilience, and IWB. Structural equation modeling further supported the hypothesis that CQ partially mediates the relationship between resilience and IWB. This mediating effect implies that resilient nurses are more likely to exhibit innovative behaviors when they possess high cultural intelligence. These findings echo prior research by Robinson et al. (2024), Özçetin and Sarıoğlu (2021), and Lee et al. (2018), who identified CQ as a facilitator of both personal adaptation and professional performance [38, 68, 69]. The role of CQ in enhancing innovation is further emphasized in studies by Afsar et al. (2021), Fan et al. (2020), and Korzilius et al. (2017) [70–73]. However, Nurlaela et al. (2022) and So et al. (2024) found that CQ does not influence IWB [74, 75], suggesting the influence of contextual variables such as organizational support or individual creativity.

To broaden this discussion, Agaoglu et al. (2025) provided insights into the complex interplay of transformational leadership, AI perceptions, and employee happiness in fostering IWB in nurses. Their findings underscore the need for multilevel approaches that incorporate leadership, digital transformation, and emotional well-being in understanding innovation in nursing. Integrating these variables into future models could provide a more comprehensive understanding of the dynamics that foster innovation in culturally diverse healthcare environments [76].

In summary, this study highlights the integral roles of cultural intelligence and resilience in promoting innovative work behavior among ER nurses. These findings offer actionable insights for nursing educators and healthcare managers aiming to build a resilient and innovative workforce through culturally competent training and supportive institutional practices.

### Implications of the study

This study underscores the critical role of cultural intelligence (CQ), resilience, and innovative work behavior (IWB) in enhancing the performance and adaptability of nurses in emergency care settings. The findings offer several actionable recommendations for nursing education, clinical practice, and healthcare leadership. First, healthcare organizations should integrate CQ assessments into recruitment and selection processes using standardized, validated tools to identify candidates with strong intercultural adaptability. This approach can help ensure that nursing staff are well-equipped to work effectively in diverse and dynamic clinical environments. Second, continuous professional development programs should include structured CQ training such as cultural sensitivity workshops, simulation-based learning, and experiential learning activities to enhance nurses’ competence in managing cross-cultural interactions.

Third, academic institutions should embed CQ, resilience-building strategies, and innovation-focused content within undergraduate and diploma nursing curricula. Preparing students with these competencies will better equip future nurses to navigate complex clinical challenges and promote patient-centered care across diverse populations. Finally, hospital leadership should cultivate a supportive work environment that values psychological safety, encourages innovation, and builds resilience. This can be achieved through inclusive management practices, recognition of adaptive behaviors, mentoring, and creating opportunities for staff to engage in problem-solving and reflective learning. Collectively, these strategies can enhance emergency nurses’ capacity to provide high-quality, culturally responsive care while fostering a resilient and innovative workforce.

## Conclusion

The results revealed that emergency nurses demonstrated moderate levels of cultural intelligence (mean percent score = 58.94%), resilience at work (58.40%), and innovative work behavior (61.49%). Pearson correlation analysis showed significant positive relationships between cultural intelligence and resilience (*r* = 0.504, *p* < 0.001), cultural intelligence and innovative work behavior (*r* = 0.588, *p* = 0.002), and between resilience and innovative work behavior (*r* = 0.664, *p* < 0.001), indicating that higher levels of resilience and CQ are associated with greater innovative behaviors. Path analysis confirmed that CQ partially mediates the relationship between resilience and innovative work behavior. Resilience had a moderate positive direct effect on both CQ (estimate = 0.304) and IWB (estimate = 0.228), while CQ had a smaller but significant direct effect on IWB (estimate = 0.119). The model explained 32% of the variance in CQ and 34% in IWB, supporting the mediating role of CQ. However, resilience appeared to be the stronger predictor of IWB. These findings highlight the importance of resilience and CQ in enhancing innovation among emergency nurses.

### Limitations of the study

Despite its valuable contributions, this study is subject to several methodological limitations that should be considered when interpreting the findings. First, a cross-sectional design restricts the ability to infer temporal or causal relationships among cultural intelligence, resilience, and innovative work behavior. Although structural equation modeling (SEM) allows for the examination of complex associations, it does not establish directionality or causality. Second, the use of convenience sampling from a single geographical and institutional context may limit the external validity of the findings, reducing their generalizability to broader or more diverse populations of emergency nurses. Third, reliance on self-reported measures introduces potential biases, including social desirability bias and common method variance, which may affect the accuracy of the responses. Future research should employ longitudinal designs to assess changes and causal relationships over time.

## Data Availability

The datasets used and/or analyzed during the current study are available from the corresponding author upon reasonable request.

## Abbreviations

CQ: Cultural intelligence
ERNs: Emergency care nurses
IWB: Innovative work behavior
SCT: Social Cognitive Theory
